# Supplementary material for: Experimental Airway Allogenic Transplantation Model with Decellularized Cryopreserved Tracheas
Source: Biomedicines. 2025 Sep 30;13(10):2401. doi: 10.3390/biomedicines13102401 (PMC12561892; doi:10.3390/biomedicines13102401)
Supplement: Supplementary file 1 [file biomedicines-13-02401-s001.zip › biomedicines-3789647-supplementary.pdf]

Experimental Airway Allogenic Transplantation Model with Decellularized Cryopreserved Tracheas

Néstor J. Martínez-Hernández*, Lara Milian-Medina, Jorge Mas-Estellés, Amparo Roig-Bataller, Manuel Mata-Roig

Néstor J. Martínez-Hernández, Amparo Roig-Bataller

Department of Thoracic Surgery. Hospital Universitari de la Ribera

Alzira, València 46600 Spain

Mail: nessmartinez@gmail.com

Lara Milian-Medina, Manuel Mata-Roig

Pathology Department, Medicine and Odontology Faculty, University of València

València, 46010 Spain

Mail: lara.milian@uv.es

Jorge Mas-Estellés

Biomaterials Centre, Polytechnic University of València

València, 46022 Spain

Mail: jmas@fis.upv.es

Manuel Mata-Roig

Networking Research Centre on Respiratory Diseases (CIBERER), ISCIII

Madrid, 28220 Spain

Mail: manuel.mata@uv.es

CONTROLS

Table S1. Tensile tests in control tracheas.					
	σ_{max} (N/mm^2)	ε_{max}	W/Vol (mJ/mm^3)	E (MPa)	R^2
Control 1	0.56620241	1.13925889	0.41038302	0.934928588	0.99792816
Control 2	0.43246898	0.92905255	0.15048411	0.845893859	0.9985578
Control 3	0.40420303	0.90215056	0.12909439	0.953563904	0.99875489
Control 4	0.54856126	0.81976858	0.15110825	1.368955241	0.99938136
Control 5	0.38803491	0.93763317	0.12716517	0.83060708	0.99864644
Control 6	0.53531319	0.98486181	0.20353373	1.15304876	0.9959046
Control 7	0.35974565	0.56893946	0.08668883	0.938762450	0.99874594
Control 8	0.3292476	0.67818781	0.12086441	0.80546461	0.99887459
Control 9	0.63815134	1.01073746	0.30837771	1.16485761	0.99327878
Control 10	0.48841058	0.69674901	0.15157978	1.08214574	0.99917287

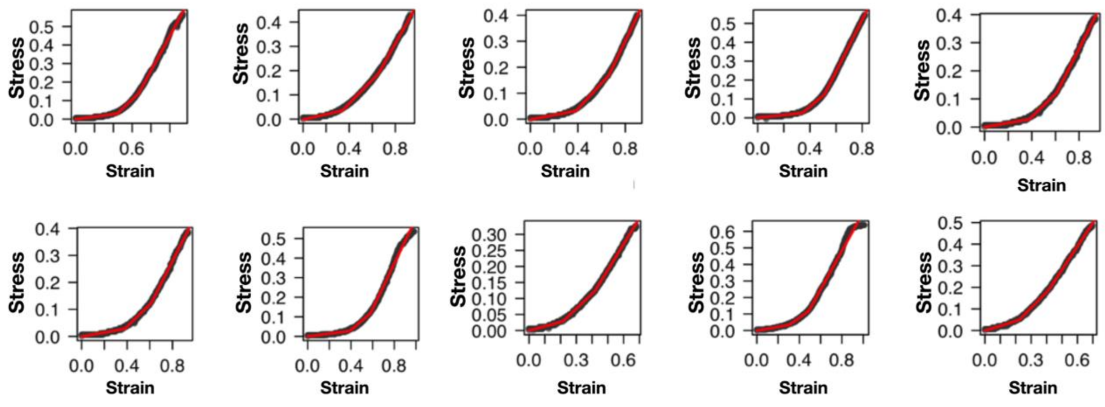

Figure S1. Stress-strain curves in tensile tests in control tracheas. On them, represented in red is the result of the segmented lineal regression to determine Young modulus.

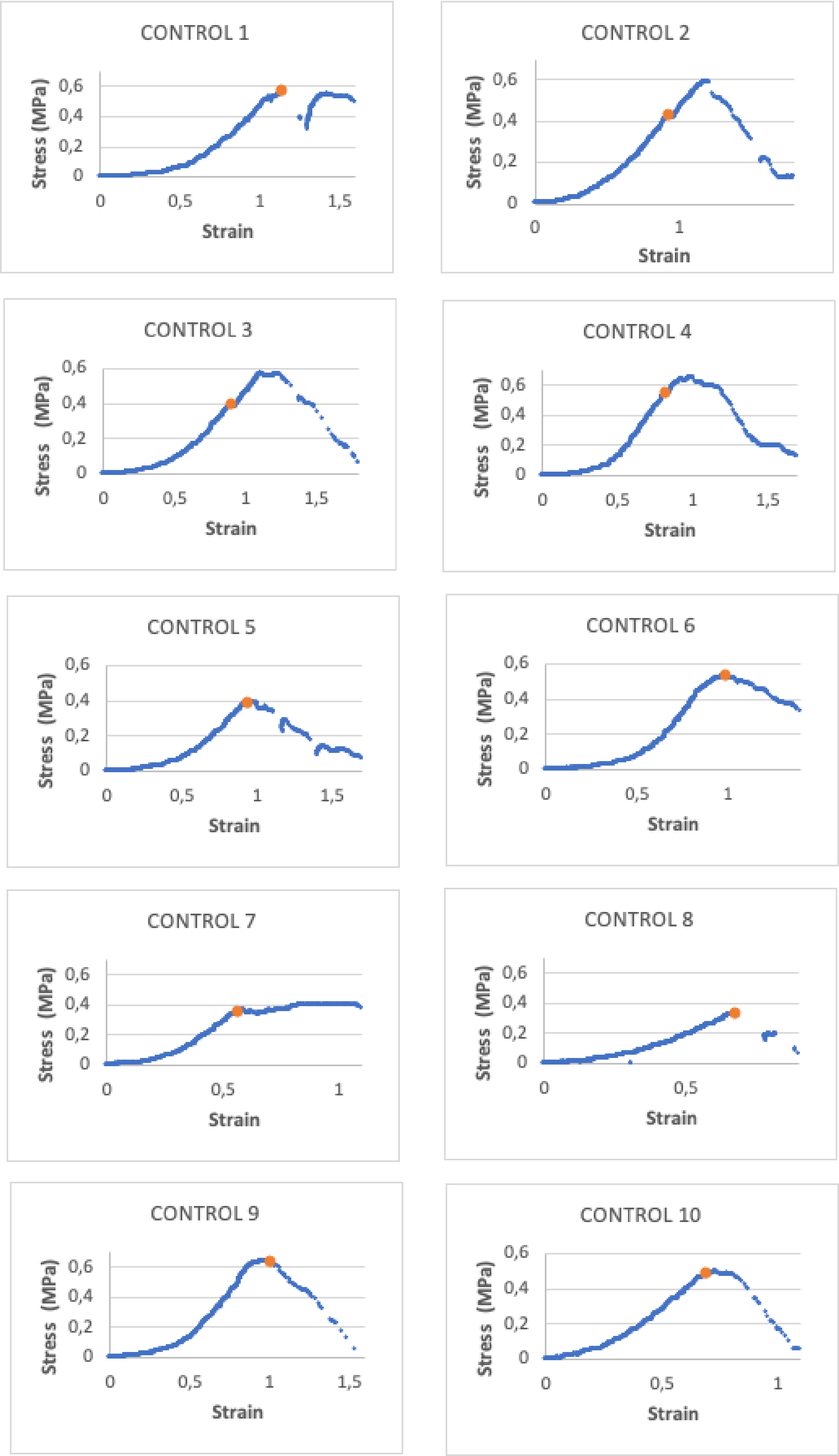

Figure S2. Stress-strain curves in tensile tests in control tracheas. The orange dot marks the breaking point.

Table S2. Compression tests in control tracheas					
		f (N/mm)	$\mathcal{R}$ $(Mpa \cdot mm)$	R^2	W/S (mJ/mm^2)
Control 1	25%	0.025101336	0.1638327	0.997012177	0.11763228
	50%	0.068802392	0.222956354	0.998417427	
	75%	0.16695744	0.719024065	0.998370916	
	100%	0.55047896	2.884581423	0.986746954	
Control 2	25%	0.01263868	0.07017383	0.91366111	0.06175561
	50%	0.03600847	0.11793468	0.98844924	
	75%	0.08392836	0.36330023	0.99356025	
	100%	0.29732534	2.4991525	0.99724707	
Control 3	25%	0.02655163	0.12965658	0.97408101	0.09201494
	50%	0.05756974	0.15144803	0.98578378	
	75%	0.1216738	0.47044318	0.99818636	
	100%	0.4415049	3.42037414	0.99662314	
Control 4	25%	0.02830126	0.11819036	0.98279867	0.0896916
	50%	0.05960658	0.14729238	0.98500466	
	75%	0.12236075	0.40243118	0.99626884	
	100%	0.4391475	4.46150808	0.99538744	
Control 5	25%	0.01756126	0.05047939	0.99219239	0.04554423
	50%	0.03210593	0.06333302	0.99822932	
	75%	0.06119527	0.18608305	0.99595152	
	100%	0.16047853	0.98869009	0.98834538	
Control 6	25%	0.01968395	0.08728295	0.99886178	0.08908877
	50%	0.05333565	0.14737916	0.99940489	
	75%	0.11624972	0.43571639	0.99537953	
	100%	0.48154151	5.01062768	0.98035939	
Control 7	25%	0.02317265	0.09371519	0.99878749	0.06643227
	50%	0.04997517	0.11725499	0.99823538	
	75%	0.0944033	0.27272928	0.99703143	
	100%	0.24036052	1.59283328	0.98042471	
Control 8	25%	0.01875986	0.09687166	0.99864408	0.06524713
	50%	0.04619364	0.12529424	0.99944351	
	75%	0.09127328	0.29904447	0.99864767	
	100%	0.25794384	1.80913788	0.97548428	
Control 9	25%	0.03080586	0.13490508	0.99933544	0.08182979
	50%	0.0644816	0.14762471	0.99894962	
	75%	0.11590537	0.31569821	0.99686068	
	100%	0.28261558	1.50499843	0.98885507	
Control 10	25%	0.00824307	0.02648697	0.975688	0.02699918
	50%	0.01976784	0.0570636	0.99390802	
	75%	0.03746661	0.10954773	0.99621241	
	100%	0.09975525	0.54118001	0.99243316	

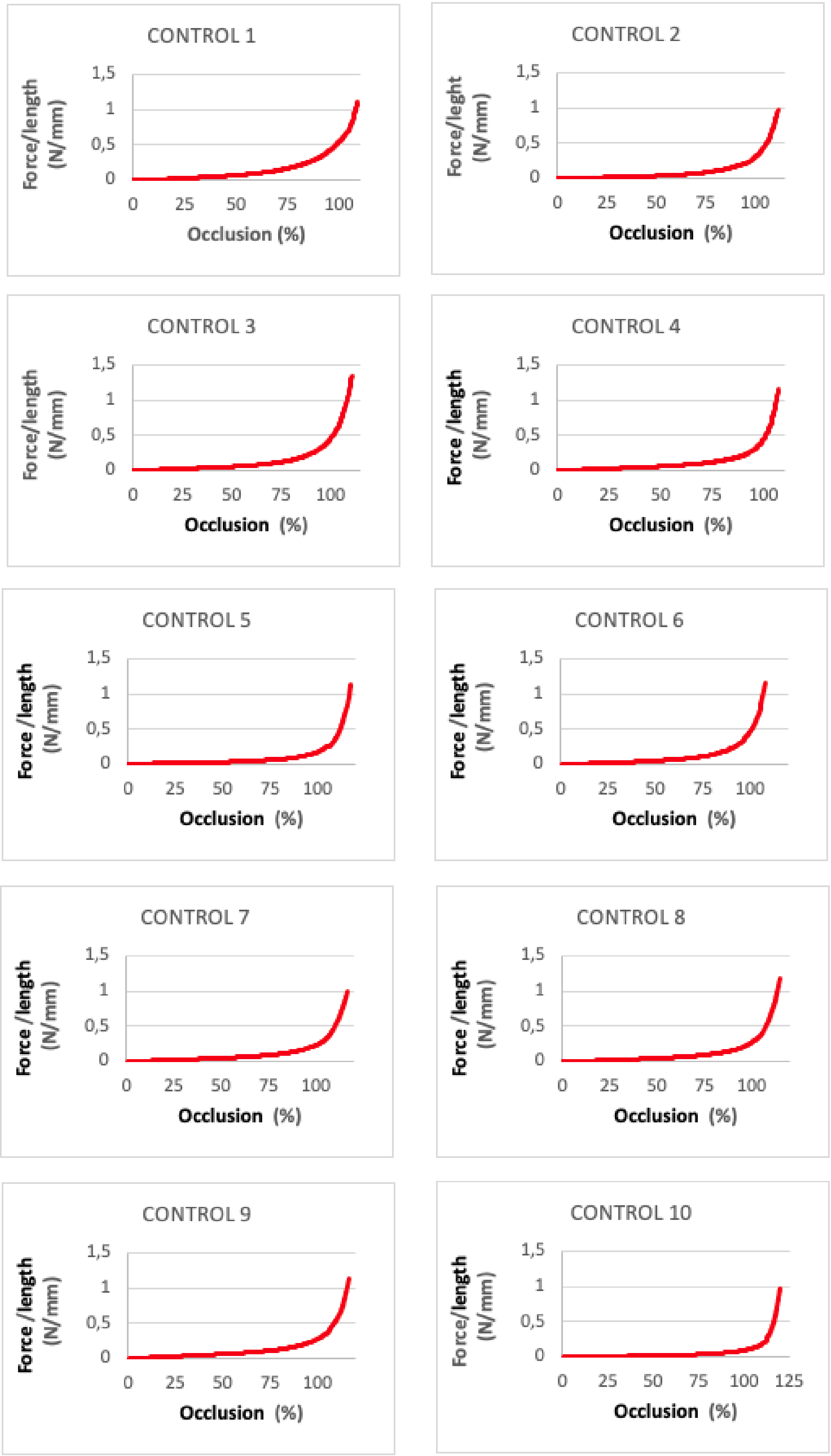

Figure S3. f per occlusion percentage curves of compression in native control tracheas

DECELLULARIZED NON-CRYOPRESERVED TRACHEAS

Table S3. Tensile tests in decellularized non-cryopreserved tracheas.

	σ_{max} (N/mm^2)	ε_{max}	W/Vol (mJ/mm^3)	E (MPa)	R^2
Decellularized non-cryopreserved 1	0.28676566	0.64012933	0.06514275	0.939079881	0.998769608
Decellularized non-cryopreserved 2	0.31351755	0.7908369	0.7908369	0.68753369	0.9983495
Decellularized non-cryopreserved 3	0.06229156	0.37147725	0.05186323	0.293344305	0.985217058
Decellularized non-cryopreserved 4	0.31695152	0.84366646	0.10844458	0.69856444	0.99726884

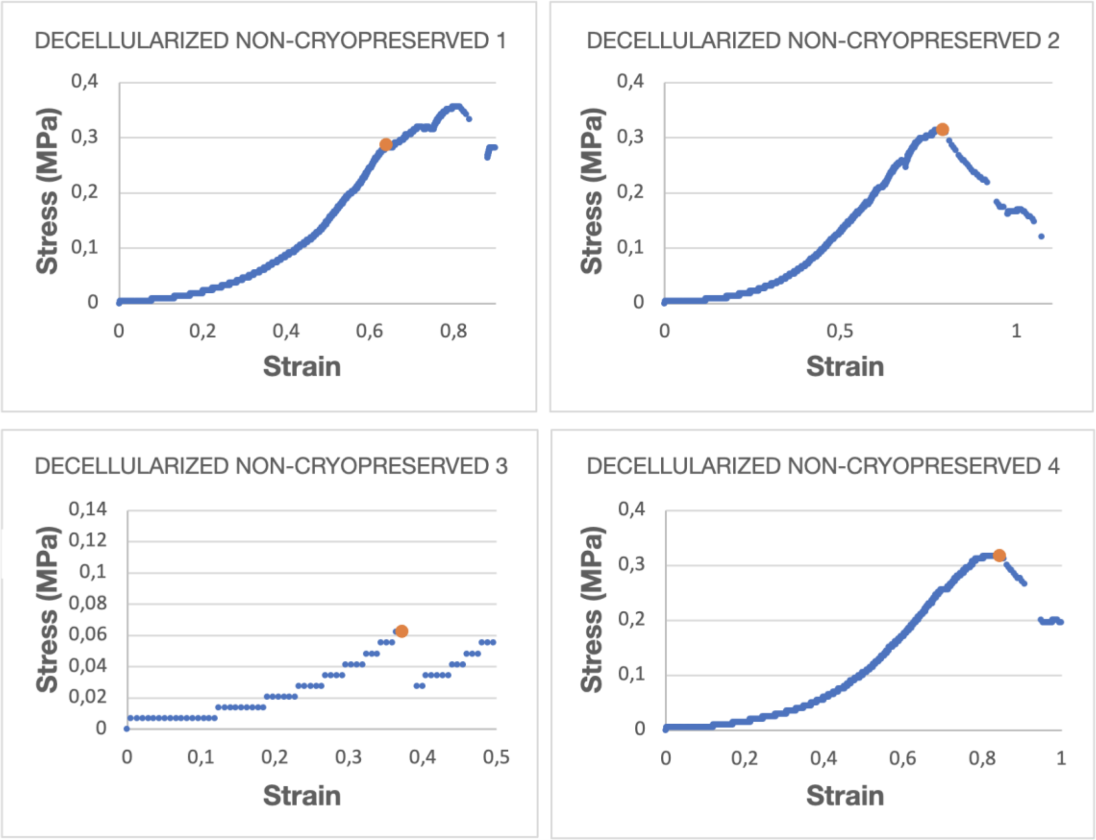

Figure S4. Stress-strain curves in tensile tests in decellularized non-cryopreserved tracheas. The orange dot marks the breaking point.

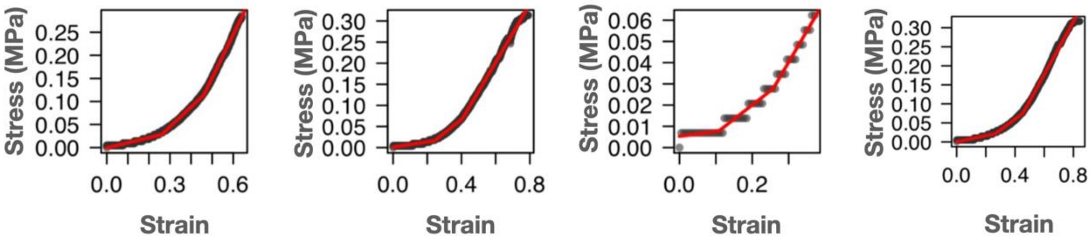

Figure S5. Stress-strain curves in tensile tests in decellularized non-cryopreserved tracheas. On them, represented in red is the result of the segmented lineal regression to determine Young modulus.

Table S4. Compression tests in decellularized non-cryopreserved tracheas					
		f $\left(^{N}/_{mm}\right)$	$\mathcal{R}$ $(Mpa \cdot mm)$	R^2	$^{W}/_{S}$ $\left(^{mJ}/_{mm^2}\right)$
Decellularized non-cryopreserved 1	25%	0.026759	0.14887163	0.99943407	0.08553667
	50%	0.06636753	0.18654313	0.99847282	
	75%	0.13298187	0.36358596	0.99879728	
	100%	0.26723933	0.8948395	0.99540105	
Decellularized non-cryopreserved 2	25%	0.03557381	0.20396071	0.99932754	0.10498227
	50%	0.09150036	0.25302057	0.99982685	
	75%	0.16544255	0.37091087	0.9994067	
	100%	0.27541573	0.56848624	0.97668508	
Decellularized non-cryopreserved 3	25%	0.00615534	0.02679152	0.99084214	0.01775436
	50%	0.01512418	0.03727378	0.9973153	
	75%	0.0257054	0.06051645	0.99203312	
	100%	0.05361967	0.19896979	0.9962536	
Decellularized non-cryopreserved 4	25%	0.01935882	0.07987446	0.99900544	0.04763896
	50%	0.04130557	0.08880933	0.99858148	
	75%	0.07089307	0.15244691	0.99932289	
	100%	0.13136858	0.44532851	0.99199854	

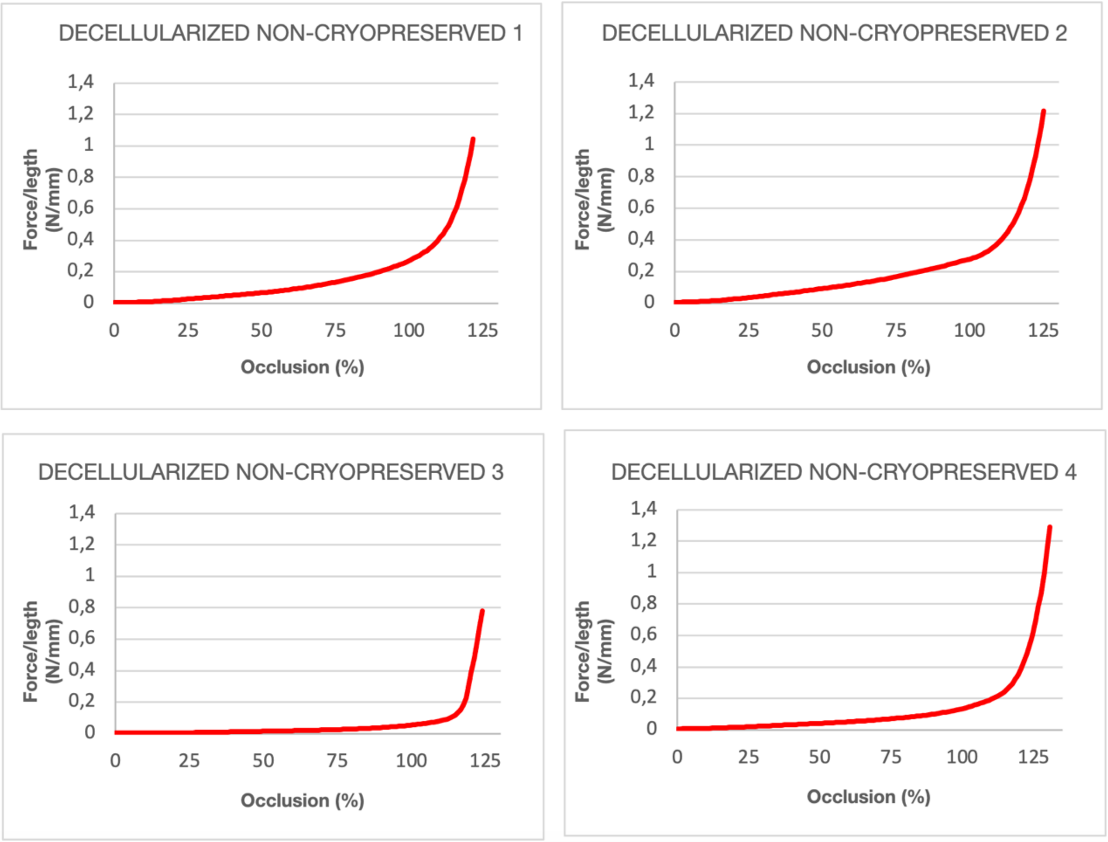

Figure S6. f per occlusion percentage curves of compression in decellularized non-cryopreserved tracheas.

DECELLULARIZED CRYOPRESERVED TRACHEAS

Table S5. Tensile tests in decellularized cryopreserved tracheas.					
	σ_{max} $({}^{N}/_{mm^2})$	ε_{max}	${}^{W}/_{Vol}$ $({}^{mJ}/_{mm^3})$	E (MPa)	R^2
Decellularized cryopreserved 1	0,23126649	0.8447467	0.08295248	0.450701404	0.997401629
Decellularized cryopreserved 2	0.07906587	0.60231885	0.02377653	0.194237888	0.995152737
Decellularized cryopreserved 3	0.25704701	0.86614847	0.08670318	0.560490726	0.99803033
Decellularized cryopreserved 4	0.45882506	0.58685413	0.12242781	1.54950156	0.998076727

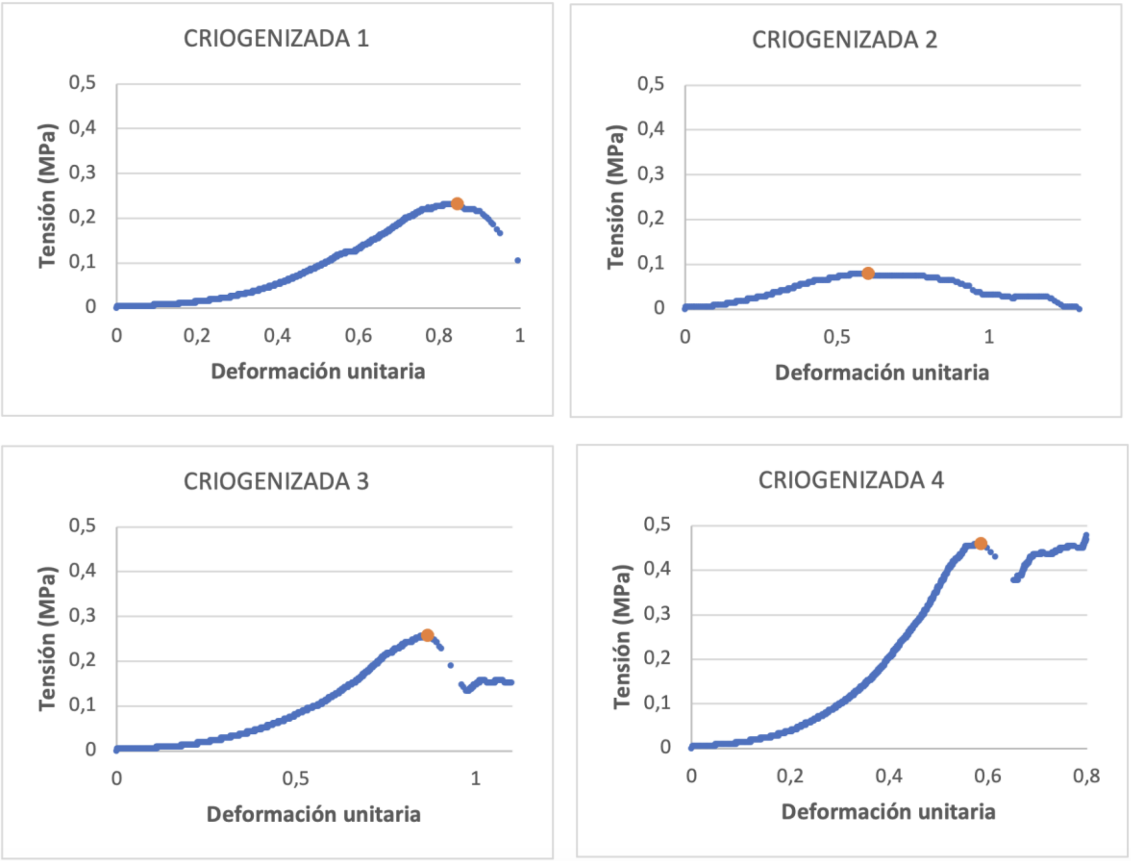

Figure S7. Stress-strain curves in tensile tests in decellularized cryopreserved tracheas. The orange dot marks the breaking point.

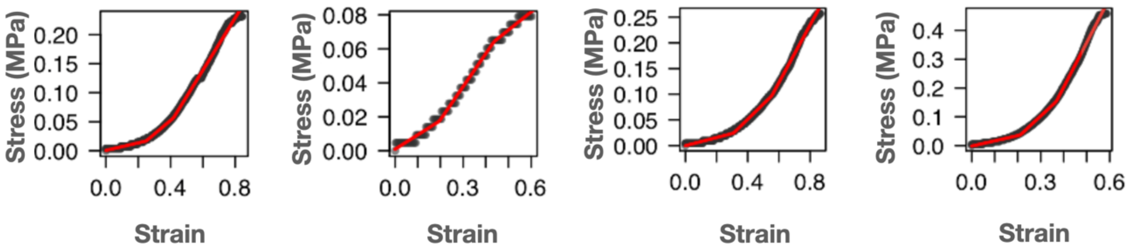

Figure S8. Stress-strain curves in tensile tests in decellularized cryopreserved tracheas. On them, represented in red is the result of the segmented lineal regression to determine Young modulus.

Table S6. Compression tests in decellularized cryopreserved tracheas					
		f $\left({}^{N}/_{mm}\right)$	$\mathcal{R}$ $(Mpa \cdot mm)$	R^2	${}^{W}/_{S}$ $\left({}^{mJ}/_{mm^2}\right)$
Decellularized cryopreserved 1	25%	0,00918811	0,01024683	0,90599479	0,01422099
	50%	0,01152998	0,01038917	0,96669188	
	75%	0,01660403	0,04111375	0,98696429	
	100%	0,04080339	0,2084726	0,98459284	
Decellularized cryopreserved 2	25%	0,00520851	0,01083867	0,95625405	0,00895111
	50%	0,0082675	0,01479881	0,97195543	
	75%	0,01204025	0,01638842	0,98380543	
	100%	0,01815821	0,04074716	0,98841792	
Decellularized cryopreserved 3	25%	0,00699906	0,01305482	0,9419434	0,01376554
	50%	0,01157826	0,01950127	0,97465106	
	75%	0,01832657	0,03969066	0,99386615	
	100%	0,0390535	0,24461473	0,97000712	
Decellularized cryopreserved 4	25%	0,03447359	0,23078059	0,99953159	0,12094272
	50%	0,09601747	0,27874796	0,99952229	
	75%	0,18117175	0,45357536	0,99904677	
	100%	0,40507942	1,9143551	0,99285479	

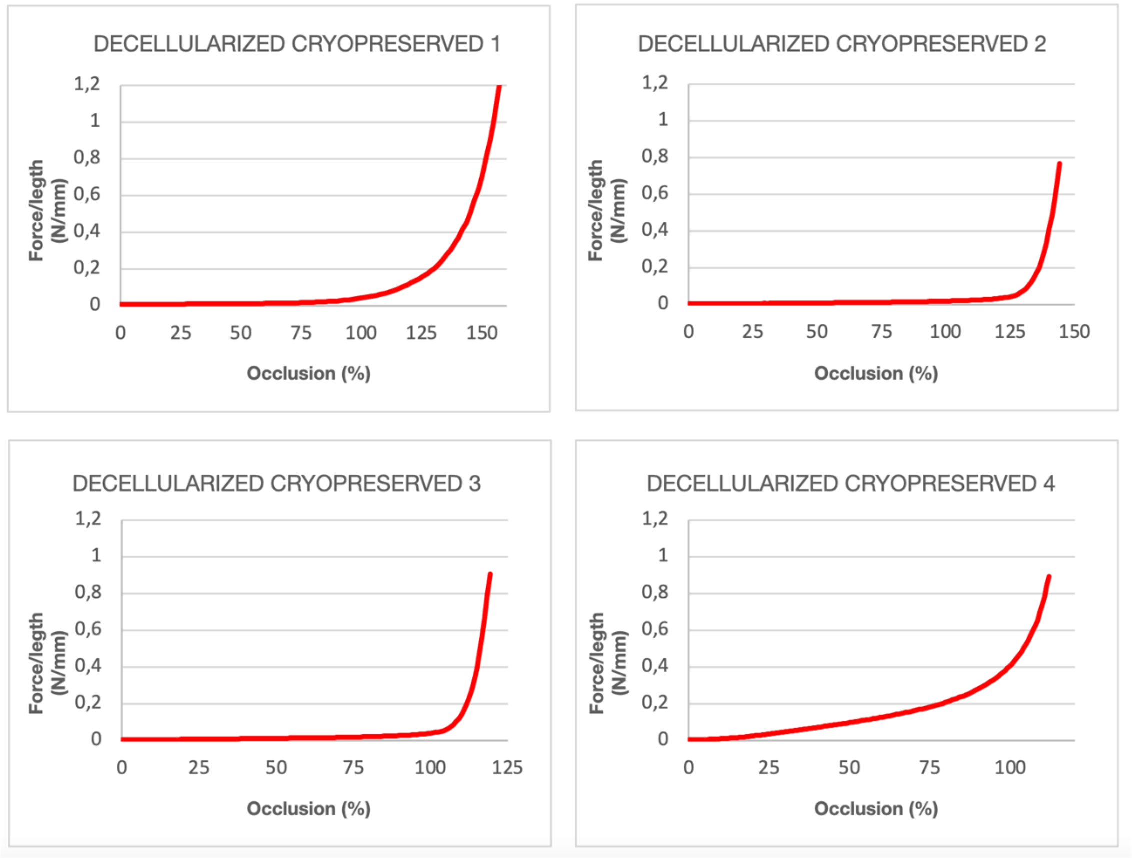

Figure S9. f per occlusion percentage curves of compression in decellularized cryopreserved tracheas.

Table S7. Histological characterization of 2 weeks implanted tracheas											
		**E**	**N**	**L**	**P**	**M**	**CG**	**C**	**F**	**N**	**V**
Rabbit 1	Cryopreserved	0	0	0	0	0	1	2	0	0	15
Rabbit 2	Non-Cryopreserved	3	1	0	1	1	1	2	1	1	11
	Cryopreserved	1	0	0	0	0	1	3	1	1	12
Rabbit 3	Non-Cryopreserved	0	0	0	0	1	1	2	1	0	17
	Cryopreserved	3	1	0	1	1	1	3	1	1	24
Rabbit 4	Non-Cryopreserved	2	1	1	2	4	3	3	2	0	5
	Cryopreserved	3	1	1	1	1	2	4	1	0	9

E: Eeosinophils; **N:** Neutrophils; **L:** Lymphocytes; **P:** Plasma cells; **M:** macrophages; **G:** Giant cells; **C:** connective tissue formation; **F:** Fat tissue; **N:** Cartilage necrosis; **V:** Vascularization

Table S8. Histological characterization of 4 weeks implanted tracheas											
		**E**	**N**	**L**	**P**	**M**	**CG**	**C**	**F**	**N**	**V**
Rabbit 5	Non-Cryopreserved	1	0	0	0	0	1	1	0	0	19
	Cryopreserved	2	1	0	1	2	0	3	1	1	24
Rabbit 6	Non-Cryopreserved	2	0	0	0	1	2	4	1	0	23
	Cryopreserved	2	1	1	2	2	1	3	1	1	21
Rabbit 7	Non-Cryopreserved	2	1	1	1	1	1	2	1	3	23
Rabbit 8	Non-Cryopreserved	2	1	0	0	2	1	3	1	1	19
	Cryopreserved	2	1	0	0	2	3	5	1	1	14

E: Eeosinophils; **N:** Neutrophils; **L:** Lymphocytes; **P:** Plasma cells; **M:** macrophages; **G:** Giant cells; **C:** connective tissue formation; **F:** Fat tissue; **N:** Cartilage necrosis; **V:** Vascularization

Table S9. Histological characterization of 8 weeks implanted tracheas											
		**E**	**N**	**L**	**P**	**M**	**CG**	**C**	**F**	**N**	**V**
Rabbit 9	Non-Cryopreserved	1	0	0	0	0	1	4	1	0	26
	Cryopreserved	1	0	0	0	1	1	5	1	1	21
Rabbit 10	Non-Cryopreserved	1	0	0	0	1	2	4	1	0	25
	Cryopreserved	1	0	0	0	1	1	4	1	0	19
Rabbit 12	Cryopreserved	2	0	0	0	1	2	4	1	0	22

E: Eeosinophils; **N:** Neutrophils; **L:** Lymphocytes; **P:** Plasma cells; **M:** macrophages; **G:** Giant cells; **C:** connective tissue formation; **F:** Fat tissue; **N:** Cartilage necrosis; **V:** Vascularization

Table S10. Histological characterization of 12 weeks implanted tracheas											
		**E**	**N**	**L**	**P**	**M**	**CG**	**C**	**F**	**N**	**V**
Rabbit 13	Non-Cryopreserved	1	0	0	0	1	1	4	1	0	15
	Cryopreserved	1	0	0	1	1	1	4	1	0	21
Rabbit 14	Non-Cryopreserved	0	0	0	0	0	1	4	1	0	20
	Cryopreserved	1	0	0	0	1	1	4	1	0	13
Rabbit 15	Non-Cryopreserved	2	1	0	1	1	3	4	1	1	11
	Cryopreserved	0	0	0	0	1	3	4	1	3	16
Rabbit 16	Non-Cryopreserved	1	0	0	0	1	3	4	1	2	12
	Cryopreserved	2	1	1	0	2	4	4	1	0	27

E: Eeosinophils; **N:** Neutrophils; **L:** Lymphocytes; **P:** Plasma cells; **M:** macrophages; **G:** Giant cells; **C:** connective tissue formation; **F:** Fat tissue; **N:** Cartilage necrosis; **V:** Vascularization

2 WEEKS IMPLANT

Table S11. Tensile tests in 2 weeks implanted tracheas.					
	σ_{max} $(^{N}/_{mm^2})$	ε_{max}	$^{W}/_{Vol}$ $(^{mJ}/_{mm^3})$	E (MPa)	R^2
Rabbit 3 Cryopreserved	0.28937262	1.98927735	0.19362009	0.346639622	0.997144191
Rabbit 4 Cryopreserved	0.04681028	1.2203508	0.03484492	0.045057671	0.987329784
Rabbit 4 Cryopreserved	0.09473509	1.17741652	0.09945595	0.106504854	0.989187647

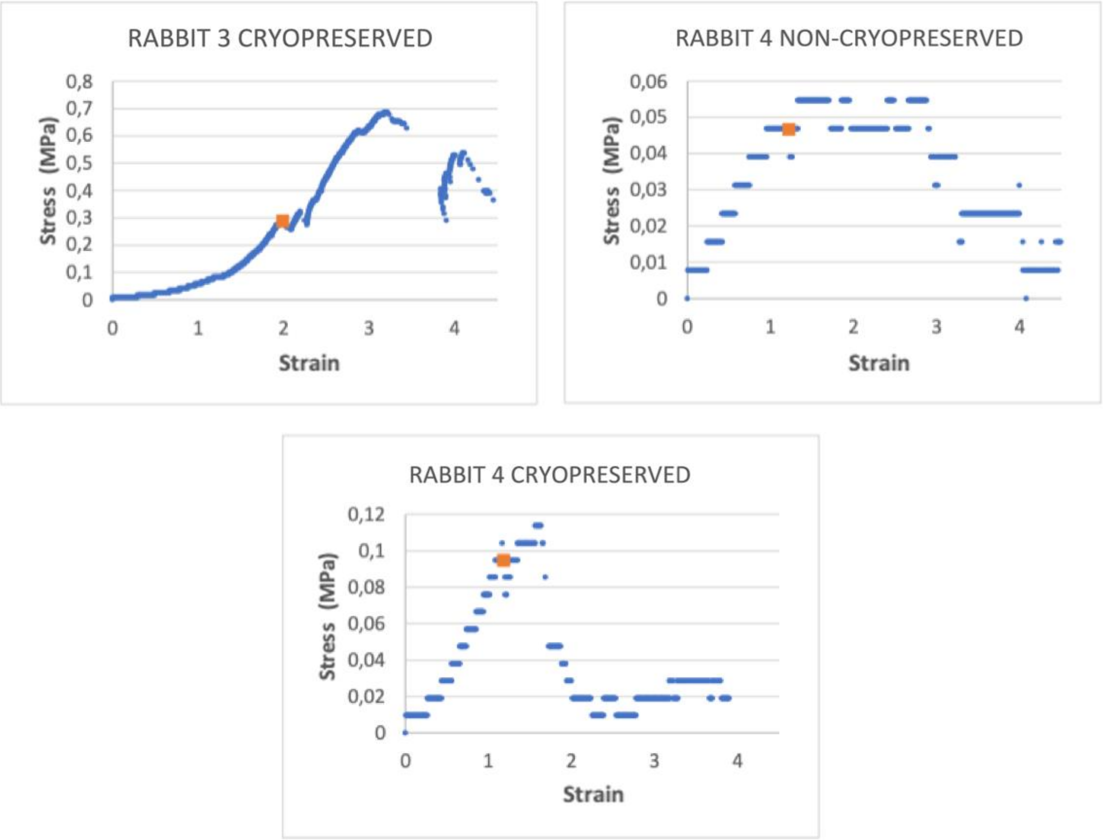

Figure S10. Stress-strain curves in tensile tests in 2 weeks implanted tracheas. The orange dot marks the breaking point (note the range change in stress in rabbit number 3).

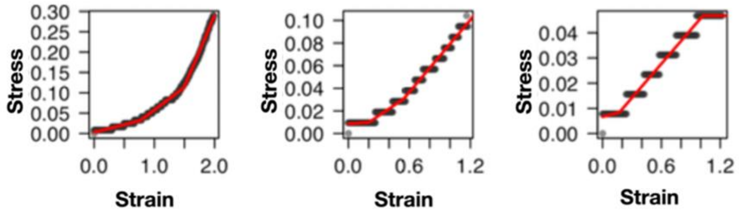

Figure S11. Stress-strain curves in tensile tests in 2 weeks implanted tracheas. On them, represented in red is the result of the segmented lineal regression to determine Young modulus.

Table S12. Compression tests in 2 weeks implanted tracheas					
		f $(^N/_{mm})$	$\mathcal{R}$ $(Mpa \cdot mm)$	R^2	$^W/_S$ $(^{mJ}/_{mm^2})$
Rabbit 3 Cryopreserved	25%	0.00904855	0.02982781	0.78509994	0.0344968
	50%	0.02163084	0.08624594	0.96273559	
	75%	0.02163084	0.15232821	0.98922917	
	100%	0.12256275	0.45448958	0.99406496	
Rabbit 4 Cryopreserved	25%	0.00894874	0.00856814	0.27717256	0.01823669
	50%	0.01264138	0.02411752	0.66986223	
	75%	0.02556559	0.0580184	0.93092645	
	100%	0.05018311	0.20588682	0.99324922	

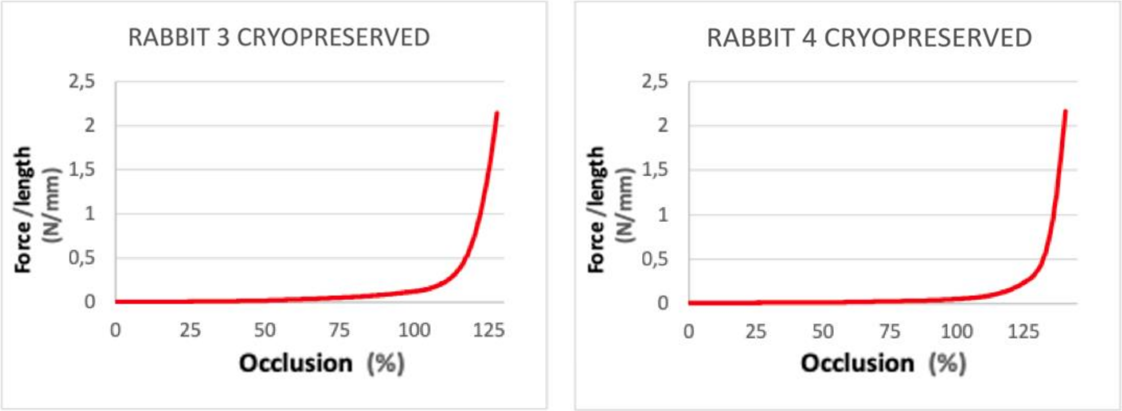

Figure S12. f per occlusion percentage curves of compression in 2 weeks implanted tracheas

4 WEEKS IMPLANT

Table S13. Tensile tests in 4 weeks implanted tracheas.					
	σ_{max} $({}^{N}/{}_{mm^2})$	ε_{max}	${}^{W}/{}_{Vol}$ $({}^{mJ}/{}_{mm^3})$	E (MPa)	R^2
Rabbit 5 Non-Cryopreserved	0.04973592	0.59329454	0.0158997	0.102086358	0.985129826
Rabbit 6 Non-Cryopreserved	0.03835059	1.25517398	0.01981013	0.048018407	0.995747686
Rabbit 6 Cryopreserved	0.11171981	1.04681181	0.06826155	0.164606655	0.996241768
Rabbit 7 Non-Cryopreserved	0.06252516	0.81044423	0.02316589	0.119372611	0.996241768
Rabbit 8 Non-Cryopreserved	0.10092752	0.81628131	0.03616252	0.211098165	0.993639759

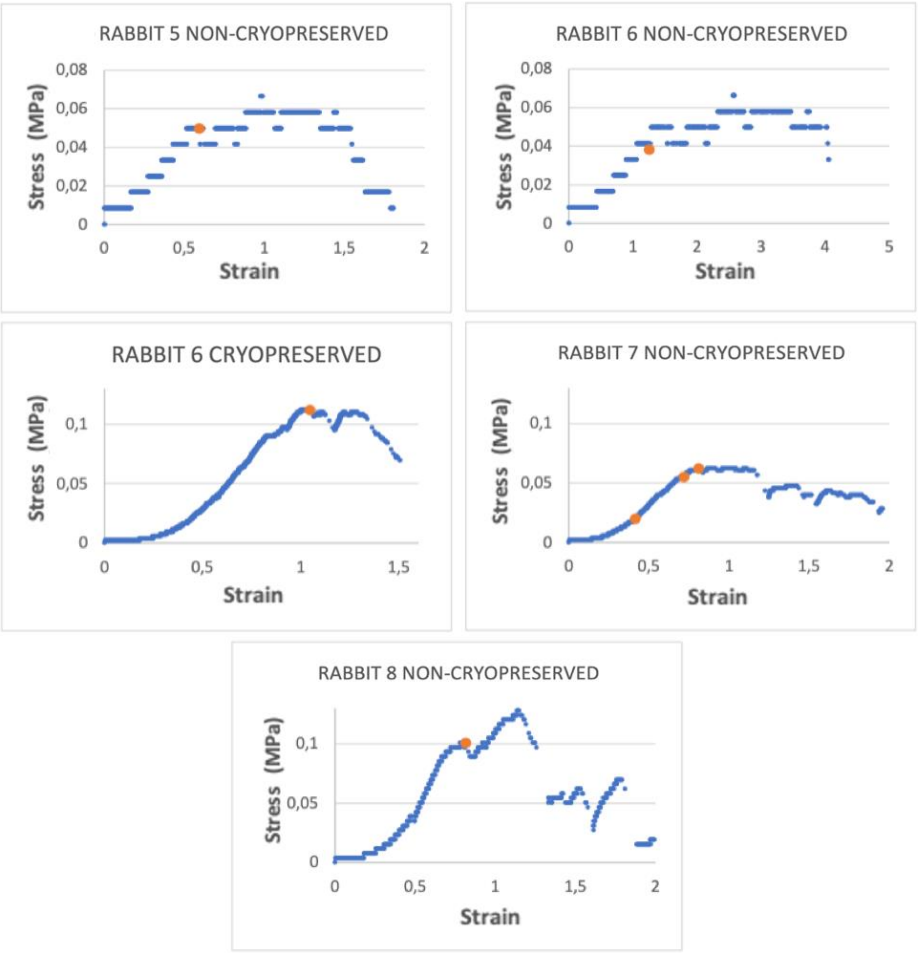

Figure S13. Stress-strain curves in tensile tests in 4 weeks implanted tracheas. The orange dot marks the breaking point.

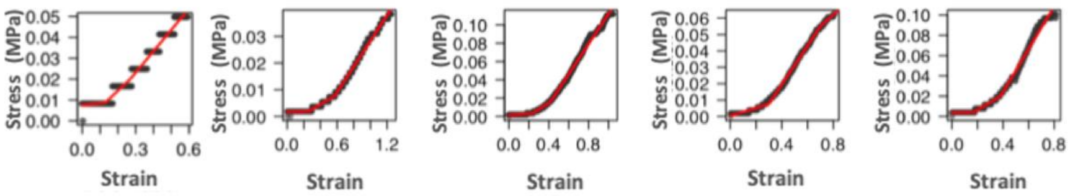

Figure S14. Stress-strain curves in tensile tests in 4 weeks implanted tracheas. On them, represented in red is the result of the segmented lineal regression to determine Young modulus.

Table S14. Compression tests in 4 weeks implanted tracheas					
		f $(^{N}/_{mm})$	$\mathcal{R}$ $(Mpa \cdot mm)$	R^2	$^{W}/_{S}$ $(^{mJ}/_{mm^2})$
Rabbit 5 Non-Cryopreserved	25%	0.01066394	0.00980549	0.76191744	0.02069396
	50%	0.01497201	0.02668037	0.95590389	
	75%	0.02510866	0.058846	0.95695755	
	100%	0.06818937	0.45865191	0.92319844	
Rabbit 6 Cryopreserved	25%	0.01030626	0.00435428	0.6138508	0.01334003
	50%	0.01197388	0.00806192	0.87882604	
	75%	0.0147533	0.01918409	0.94823928	
	100%	0.02336944	0.05606047	0.986716	
Rabbit 8 Non-Cryopreserved	25%	0.01534854	0.01011181	0.73298503	0.0233567
	50%	0.01917791	0.03446594	0.89203428	
	75%	0.02990022	0.06042347	0.9882425	
	100%	0.05057896	0.11331686	0.95405855	

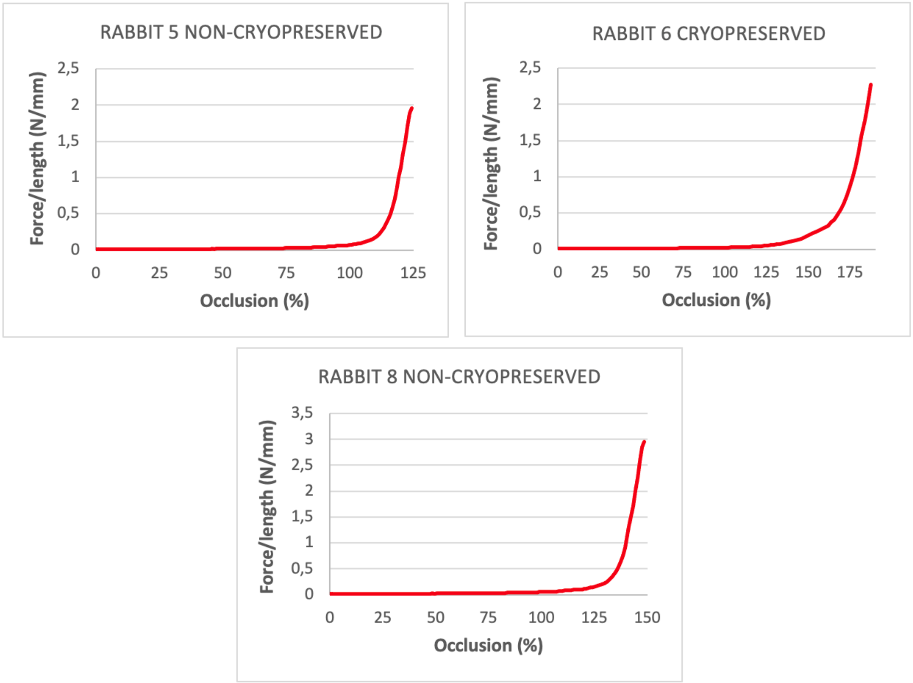

Figure S15. f per occlusion percentage curves of compression in 4 weeks implanted tracheas

8 WEEKS IMPLANT

Table S15. Tensile tests in 8 weeks implanted tracheas.					
	σ_{max} $(^{N}/_{mm^2})$	ε_{max}	$^{W}/_{Vol}$ $(^{mJ}/_{mm^3})$	E (MPa)	R^2
Rabbit 9 Non-Cryopreserved	0.20004392	0.65733344	0.0632104	0.470138101	0.998133219
Rabbit 9 Cryopreserved	0.179256067	0.930716498	0.09184159	0.333182999	0.998642675
Rabbit 10 Non-Cryopreserved	0.09983414	0.62044159	0.03090374	0.30688755	0.998091435
Rabbit 10 Cryopreserved	0.16516702	0.73455142	0.06391335	0.258368296	0.998164273

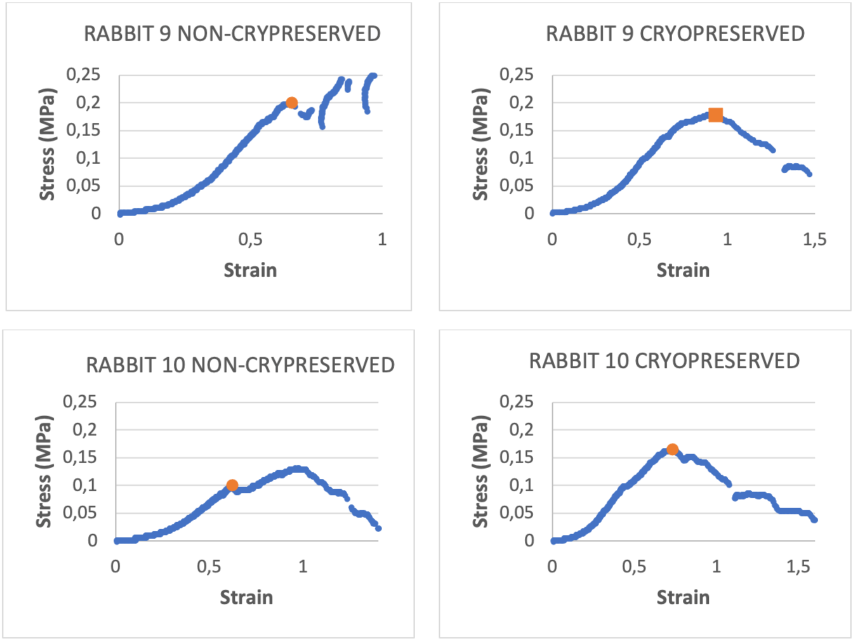

Figure S16. Stress-strain curves in tensile tests in 8 weeks implanted tracheas. The orange dot marks the breaking point.

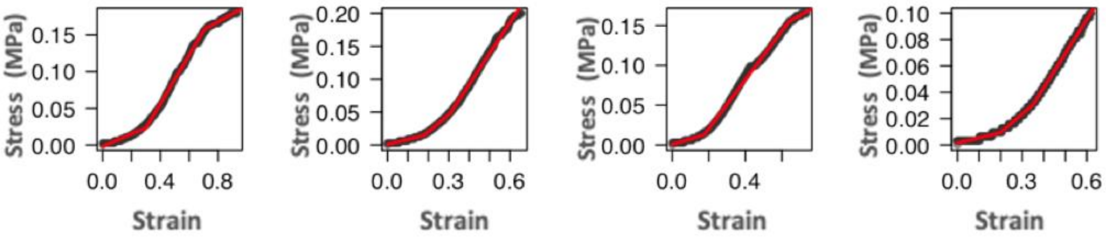

Figure S17. Stress-strain curves in tensile tests in 8 weeks implanted tracheas. On them, represented in red is the result of the segmented lineal regression to determine Young modulus.

Table S16. Compression tests in 8 weeks implanted tracheas					
		f $\left({}^{N}/_{mm}\right)$	$\mathcal{R}$ $(Mpa \cdot mm)$	R^2	${}^{W}/_{S}$ $\left({}^{mJ}/_{mm^2}\right)$
Rabbit 9 Cryopreserved	25%	0.01420633	0.02634919	0.97596192	0.02443867
	50%	0.02182428	0.03252074	0.96935183	
	75%	0.03259444	0.0611875	0.98700623	
	100%	0.05492285	0.1488456	0.99751779	
Rabbit 10 Non- Cryopreserved	25%	0.01136877	0.00232024	0.39731057	0.01169417
	50%	0.01189658	0.00129607	0.48508218	
	75%	0.01189658	0.00237464	0.50600193	
	100%	0.01321602	0.00550746	0.82103283	
Rabbit 12 Non- Cryopreserved	25%	0.00987098	0.00241018	0.27281662	0.01132847
	50%	0.01096577	0.00525838	0.85373256	
	75%	0.01249853	0.00372432	0.6306408	
	100%	0.0149071	0.0145308	0.9515862	

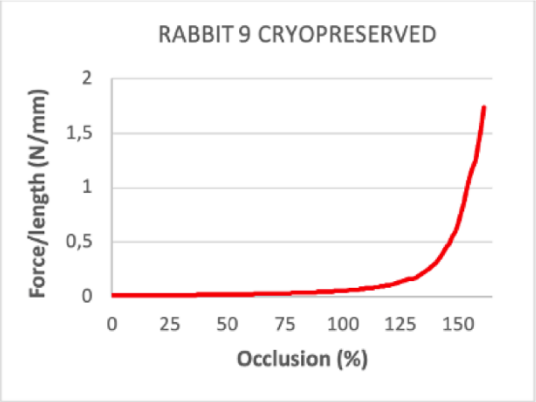

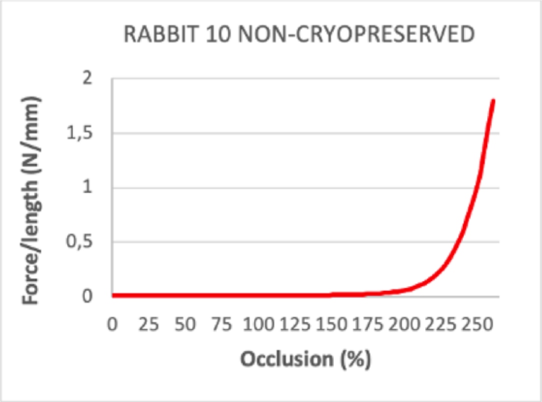

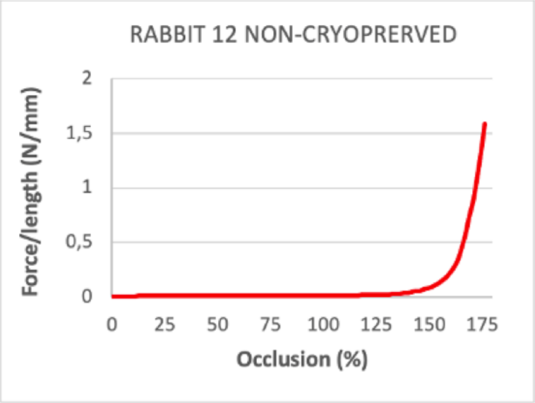

Figure S18. f per occlusion percentage curves of compression in 8 weeks implanted tracheas

12 WEEKS IMPLANT

Table S17. Tensile tests in 12 weeks implanted tracheas.					
	σ_{max} (N/mm^2)	ε_{max}	W/Vol (mJ/mm^3)	E (MPa)	R^2
Rabbit 13 Non-Cryopreserved	0.12244706	0.46786191	0.02434828	0.355406481	0.996652659
Rabbit 14 Non-Cryopreserved	0.1996741	0.5137817	0.05898284	0.507115374	0.99776739
Rabbit 14 Cryopreserved	0.15003294	0.38249353	0.02408144	0.706680292	0.99711113
Rabbit 15 Cryopreserved	0.13262912	0.59324289	0.04037654	0.322842727	0.997213748
Rabbit 16 Non-Cryopreserved	0.24665122	0.59697982	0.08250647	0.581347171	0.998486172
Rabbit 16 Cryopreserved	0.1489497	0.53762117	0.04078089	0.397055228	0.998390263

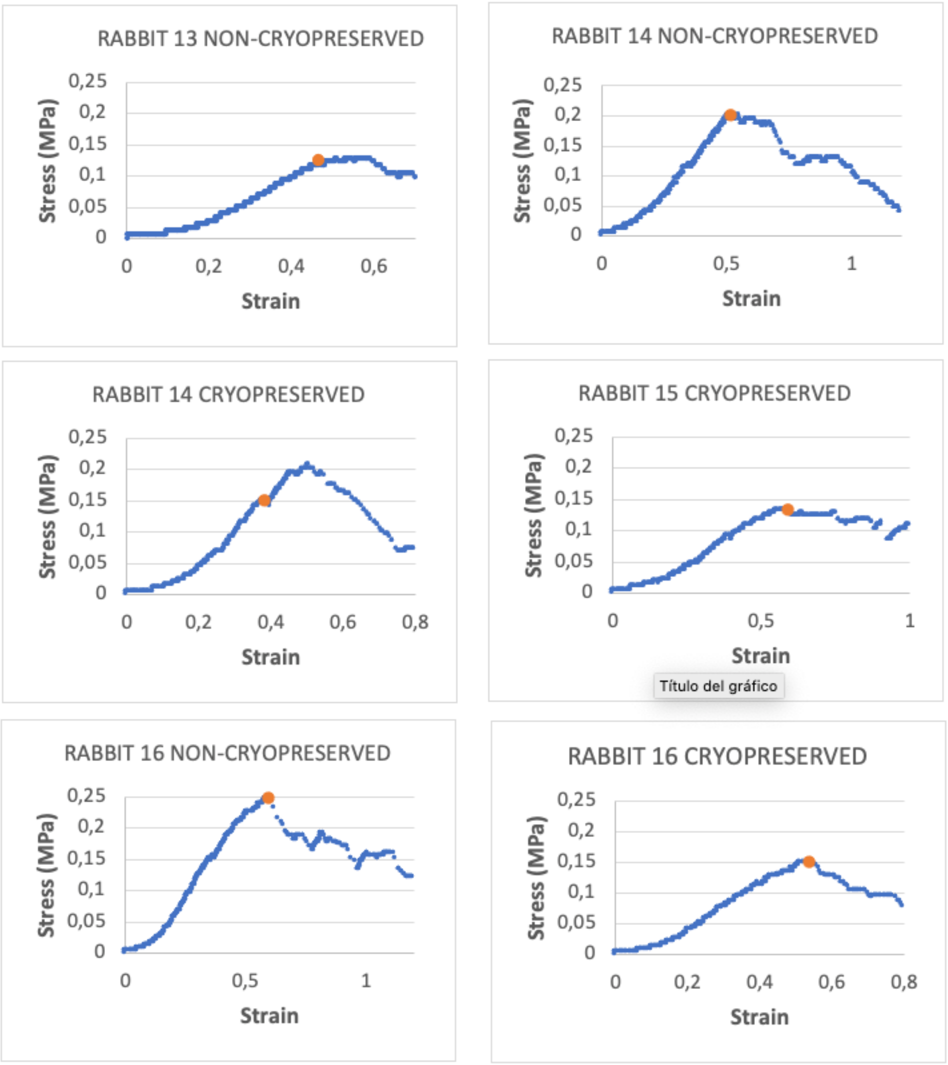

Figure S19. Stress-strain curves in tensile tests in 12 weeks implanted tracheas. The orange dot marks the breaking point.

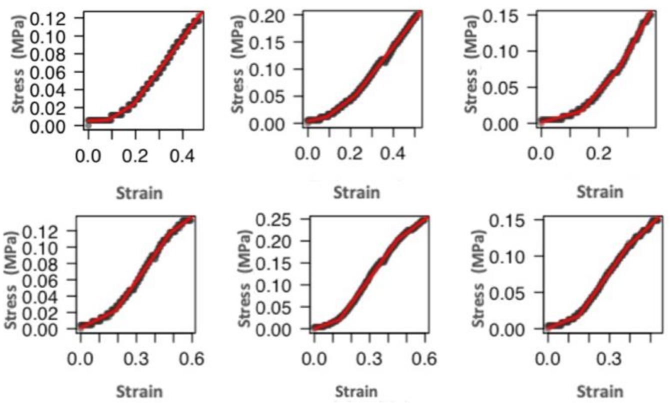

Figure S20. Stress-strain curves in tensile tests in 12 weeks implanted tracheas. On them, represented in red is the result of the segmented lineal regression to determine Young modulus.

Table S18. Compression tests in 12 weeks implanted tracheas					
		f $(^{N}/_{mm})$	$\mathcal{R}$ $(Mpa \cdot mm)$	R^2	$^{W}/_{S}$ $(^{mJ}/_{mm^2})$
Rabbit 13 Non-Cryopreserved	25%	0.00737414	0.00616387	0.75030616	0.01104251
	50%	0.00898965	0.00981303	0.91861066	
	75%	0.01347723	0.03066624	0.97558511	
	100%	0.02532443	0.07024379	0.99563997	
Rabbit 14 Non-Cryopreserved	25%	0.0094392	0.01359226	0.97105731	0.01728061
	50%	0.01383625	0.02286227	0.96240222	
	75%	0.0222116	0.04444526	0.97521914	
	100%	0.05005961	0.37728402	0.94488477	
Rabbit 14 Cryopreserved	25%	0.00916916	0.01653136	0.96895342	0.01903449
	50%	0.01347723	0.02405369	0.97433596	
	75%	0.0238884	0.07225147	0.99715359	
	100%	0.06481506	0.35629116	0.98826938	
Rabbit 15 Cryopreserved	25%	0.00552485	0.00164527	0.91298216	0.00597116
	50%	0.00601118	0.001917	0.90374051	
	75%	0.00617329	0.00221031	0.86427396	
	100%	0.00682171	0.00235937	0.73500187	
Rabbit 16 Non-Cryopreserved	25%	0.00982481	0.00860443	0.94634295	0.01376483
	50%	0.01325655	0.01457134	0.9081013	
	75%	0.01693341	0.02280095	0.92762209	
	100%	0.02477743	0.04991898	0.98817239	
Rabbit 16 Cryopreserved	25%	0.00618202	0.0014357	0.35928218	0.00638101
	50%	0.00618202	0.0012049	0.36597104	
	75%	0.00672619	0.00143647	0.5943547	
	100%	0.00745176	0.00389351	0.95454971	

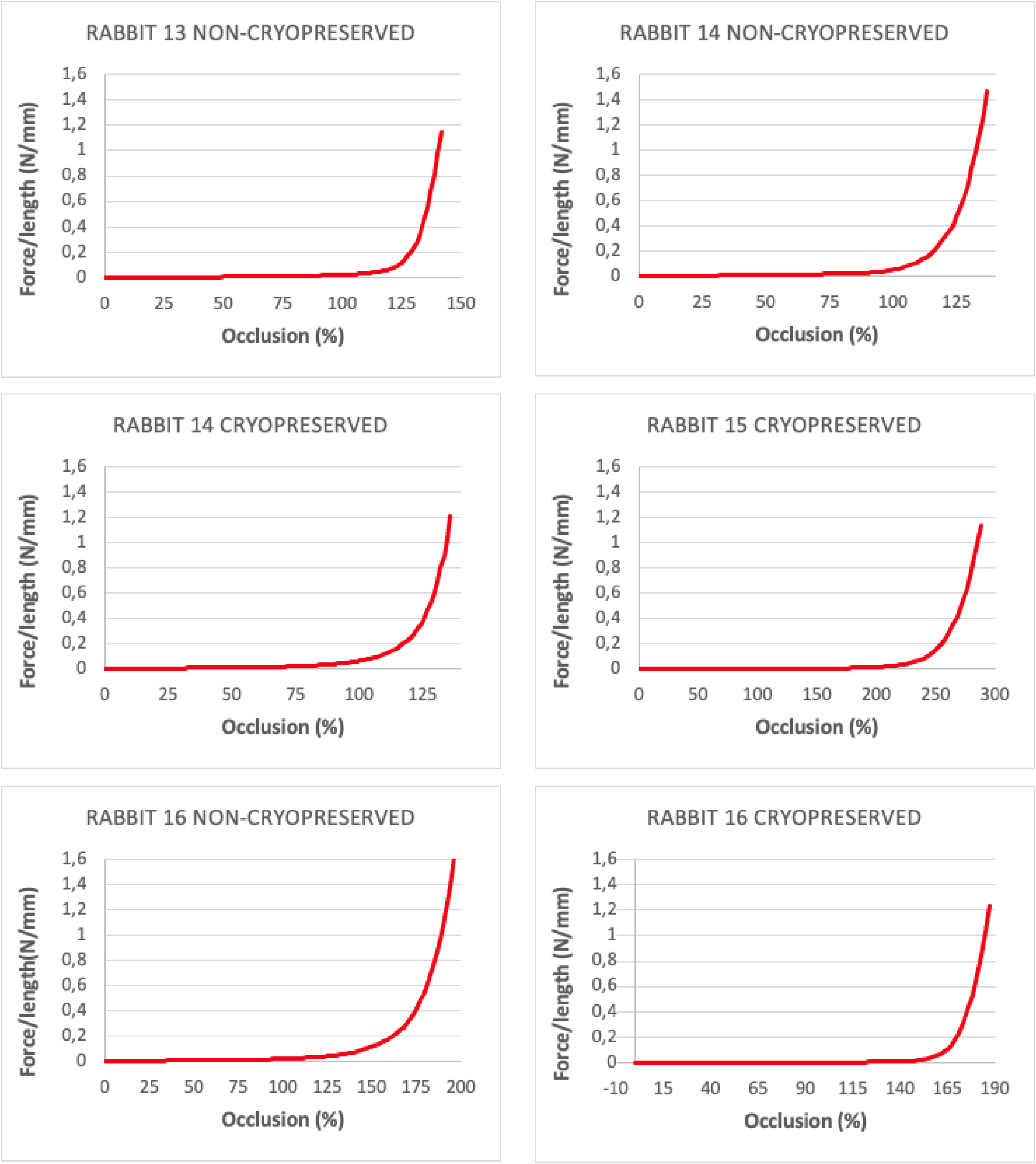

Figure S21. f per occlusion percentage curves of compression in 12 weeks implanted tracheas
